# CRISPR/Cas9-mediated gene editing in human tripronuclear zygotes

**DOI:** 10.1007/s13238-015-0153-5

**Published:** 2015-04-18

**Authors:** Puping Liang, Yanwen Xu, Xiya Zhang, Chenhui Ding, Rui Huang, Zhen Zhang, Jie Lv, Xiaowei Xie, Yuxi Chen, Yujing Li, Ying Sun, Yaofu Bai, Zhou Songyang, Wenbin Ma, Canquan Zhou, Junjiu Huang

**Affiliations:** Guangdong Province Key Laboratory of Reproductive Medicine, the First Affiliated Hospital, and Key Laboratory of Gene Engineering of the Ministry of Education, School of Life Sciences, Sun Yat-sen University, Guangzhou, 510275 China

**Keywords:** CRISPR/Cas9, β-thalassemia, human tripronuclear zygotes, gene editing, homologous recombination, whole-exome sequencing

## Abstract

Genome editing tools such as the clustered regularly interspaced short palindromic repeat (CRISPR)-associated system (Cas) have been widely used to modify genes in model systems including animal zygotes and human cells, and hold tremendous promise for both basic research and clinical applications. To date, a serious knowledge gap remains in our understanding of DNA repair mechanisms in human early embryos, and in the efficiency and potential off-target effects of using technologies such as CRISPR/Cas9 in human pre-implantation embryos. In this report, we used tripronuclear (3PN) zygotes to further investigate CRISPR/Cas9-mediated gene editing in human cells. We found that CRISPR/Cas9 could effectively cleave the endogenous β-globin gene (*HBB*). However, the efficiency of homologous recombination directed repair (HDR) of *HBB* was low and the edited embryos were mosaic. Off-target cleavage was also apparent in these 3PN zygotes as revealed by the T7E1 assay and whole-exome sequencing. Furthermore, the endogenous delta-globin gene (*HBD*), which is homologous to *HBB*, competed with exogenous donor oligos to act as the repair template, leading to untoward mutations. Our data also indicated that repair of the* HBB* locus in these embryos occurred preferentially through the non-crossover HDR pathway. Taken together, our work highlights the pressing need to further improve the fidelity and specificity of the CRISPR/Cas9 platform, a prerequisite for any clinical applications of CRSIPR/Cas9-mediated editing.

**Electronic supplementary material:**

The online version of this article (doi:10.1007/s13238-015-0153-5) contains supplementary material, which is available to authorized users.

## INTRODUCTION

The CRISPR/Cas9 RNA-endonuclease complex, consisting of the Cas9 protein and the guide RNA (gRNA) (~99 nt), is based on the adaptive immune system of *streptococcus pyogenes* SF370. It targets genomic sequences containing the tri-nucleotide protospacer adjacent motif (PAM) and complementary to the gRNA, and can be programmed to recognize virtually any genes through the manipulation of gRNA sequences (Cho et al., 2013; Cong et al., 2013; Jinek et al., 2012; Jinek et al., 2013; Mali et al., 2013c). Following Cas9 binding and subsequence target site cleavage, the double strand breaks (DSBs) generated are repaired by either non-homologous end joining (NHEJ) or homologous recombination directed repair (HDR), resulting in indels or precise repair respectively (Jinek et al., 2012; Moynahan and Jasin, 2010). The ease, expedience, and efficiency of the CRISPR/Cas9 system have lent itself to a variety of applications, including genome editing, gene function investigation, and gene therapy in animals and human cells (Chang et al., 2013; Cho et al., 2013; Cong et al., 2013; Friedland et al., 2013; Hsu et al., 2014; Hwang et al., 2013; Ikmi et al., 2014; Irion et al., 2014; Jinek et al., 2013; Li et al., 2013a; Li et al., 2013b; Long et al., 2014; Ma et al., 2014; Mali et al., 2013c; Niu et al., 2014; Smith et al., 2014a; Wu et al., 2013; Wu et al., 2014b; Yang et al., 2013).

The specificity of CRISPR/Cas9 is largely dictated by PAM and the 17–20 nt sequence at the 5′ end of gRNAs (Cong et al., 2013; Hsu et al., 2013; Mali et al., 2013a; Mali et al., 2013c; Pattanayak et al., 2013; Wu et al., 2014a). Up to 5 mismatches may be tolerated for target recognition in human cancer cells (Fu et al., 2013). Unintended mutation in the genome can greatly hinder the application of CRISPR/Cas9, especially in studies of development and gene therapy (Hsu et al., 2014; Mali et al., 2013b; Sander and Joung, 2014). Interestingly, three groups recently found through whole genome sequencing that off-target effects of CRISPR/Cas9 appeared rare in human pluripotent stem cells (Smith et al., 2014b; Suzuki et al., 2014; Veres et al., 2014), raising the possibility that high frequencies of unintended targeting by CRISPR/Cas9 may be more prevalent in cancer cell lines. Additionally, lower rates of off-target effects (compared to human cell lines) have also been reported in mouse zygotes (Wu et al., 2013; Yang et al., 2013). Despite great progress in understanding the utilization of CRISPR/Cas9 in a variety of model organisms, much remains to be learned regarding the efficiency and specificity of CRISPR/Cas9-mediated gene editing in human cells, especially in embryos. Because ethical concerns preclude studies of gene editing in normal embryos, we decided to use tripronuclear (3PN) zygotes, which have one oocyte nucleus and two sperm nuclei.

Extensive studies have shown that polyspermic zygotes such as tripronuclear (3PN) zygotes, discarded in clinics, may serve as an alternative for studies of normal human zygotes (Balakier, 1993). Polyspermic zygotes, which occur in ~2%–5% of zygotes during *in vitro* fertilization (IVF) clinical trials, may generate blastocysts *in vitro* but invariably fail to develop normally *in vivo* (Munne and Cohen, 1998), providing an ideal model system to examine the targeting efficiency and off-target effects of CRISPR/Cas9 during early human embryonic development (Bredenoord et al., 2008; Sathananthan et al., 1999).

Here, we report that the CRISPR/Cas9 system can cleave endogenous gene efficiently in human tripronuclear zygotes, and that the DSBs generated by CRISPR/Cas9 cleavage are repaired by NHEJ and HDR. Repair template of HDR can be either the endogenous homologous gene or exogenous DNA sequence. This competition between exogenous and endogenous sequence complicates the analysis of possible gene editing outcomes make it difficult to predict the consequence of gene editing. Furthermore, mosaicism and mutations at non-target sites are apparent in the edited embryos. Taken together, our data underscore the need to more comprehensively understand the mechanisms of CRISPR/Cas9-mediated genome editing in human cells, and support the notion that clinical applications of the CRISPR/Cas9 system may be premature at this stage.

## RESULTS

### CRISPR/Cas9-mediated editing of *HBB* gene in human cells

The human β-globin (*HBB*) gene, which encodes a subunit of the adult hemoglobin and is mutated in β-thalassemia (Hill et al., 1962). In China, CD14/15, CD17, and CD41/42, which are frame-shift or truncated mutations of β-globin, are three of the most common β-thalassemia mutations (Cao and Galanello, 2010). Located on chromosome 11, *HBB* is within the β-globin gene cluster that contains four other globin genes with the order of (from 5′ to 3′) *HBE*, *HBG2*, *HBG1*, *HBD*, and *HBB* (Schechter, 2008). Because the sequences of *HBB* and *HBD* are very similar, *HBD* may also be used as a template to repair *HBB*. The *HBD* footprints left in the repaired *HBB* locus should enable us to investigate whether and how endogenous homologous sequences may be utilized as HDR templates, information that will prove invaluable to any future endeavors that may employ CRISPR/Cas9 to repair gene loci with repeated sequences.

**Figure 1:**
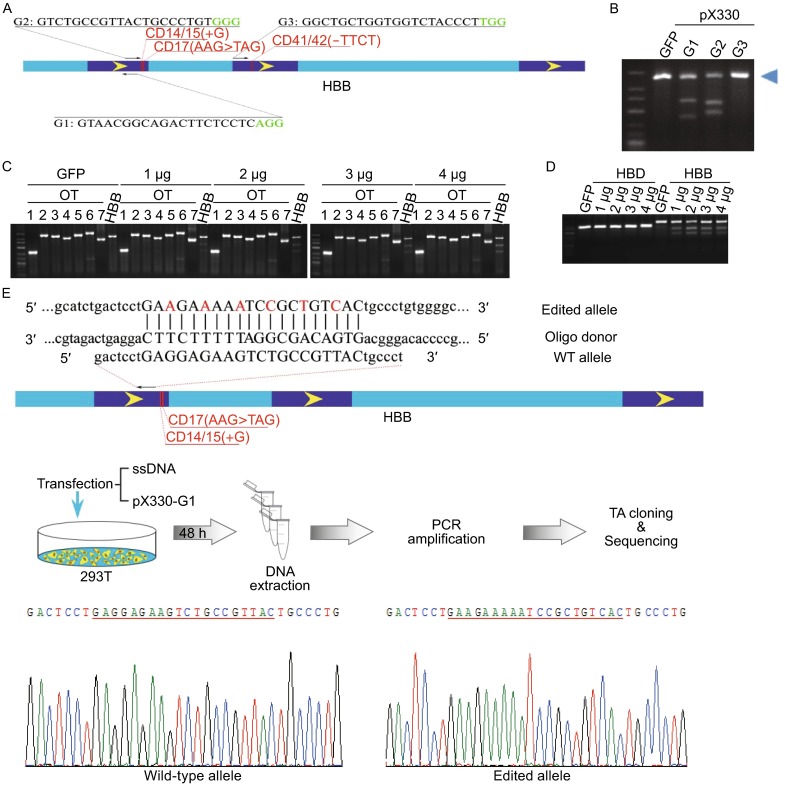
**Targeting of the** ***HBB*** **gene in human cells using CRISPR/Cas9**. (A) Three gRNA targeting sites were selected for the *HBB* locus, and the sequence for each gRNA (G1, G2, and G3) is shown with the PAM sequence in green. The three common* HBB* mutations found in β-thalassemia are indicated in red. Exons are represented by deep blue boxes with yellow arrows indicating transcriptional direction. (B) 293T cells were individually transfected with the three gRNA-Cas9 expression vectors and harvested for genomic DNA isolation 48 h after transfection. A GFP expression vector was used as transfection control. The regions spanning the gRNA target sites were then PCR amplified for the T7E1 assay. Blue arrowhead indicates the expected size for uncut (no mismatch) PCR products. (C) 293T cells were transfected with increasing concentrations (1 μg, 2 μg, 3 μg, 4 μg) of the G1 gRNA-Cas9 vector. A GFP expression vector was used as transfection control. Regions spanning the top 7 predicted off-target sites for each gRNA were PCR amplified for the T7E1 assay. OT, off-target.* HBB*, on-target editing in the* HBB* gene locus. (D) The region within the HBD locus that is highly similar to the G1 gRNA-Cas9 target sequence was analyzed as in (C). (E) A ssDNA oligo (Oligo donor) encoding 6 silent mutations (indicated in red) was synthesized (top), and co-transfected with the G1 gRNA-Cas9 construct (pX330-G1) into 293T cells (middle). At 48 h after transfection, genomic DNA was extracted to PCR amplify the region spanning the G1 target site. The PCR products were then subcloned into TA cloning vectors for sequencing analysis. Representative sequencing chromatographs for wild-type and edited alleles are shown with the mutated target region underlined in red (bottom)

Using online tools developed by Feng Zhang and colleagues (http://crispr.mit.edu/), we designed and generated three gRNAs (named G1, G2, and G3) that targeted different regions of the *HBB* gene (Fig. 1A), and transfected the gRNA-Cas9 expression vectors into human 293T cells. Compared with the GFP mock vector, G1 and G2 gRNAs exhibited efficient cleavage activities as determined by the T7E1 assay (Fig. 1B) (Shen et al., 2014). Sequencing analysis of the two regions targeted by G1 and G2 revealed distinct indel spectra, reflecting different NHEJ repair preferences at these two sites (Fig. S1). CRISPR/Cas9 targeting of the β-globin locus was previously reported to have substantially high off-target activity in cultured human cells (Cradick et al., 2013). We therefore designed specific PCR primers for the top 7 predicted off-target sites in the genome for both G1 and G2 gRNAs, along with the predicted off-target site of G1 gRNA in the *HBD* gene (Table S1). We then carried out the T7E1 assay to assess the off-target effects of G1 and G2 gRNAs in human 293T cells. While G2 gRNA showed very low off-target cleavage activity in the intergenic region (G2-OT4) (Fig. S2), gRNA G1 did not exhibit detectable off-target cleavage at the top 7 predicted off-target sites (Fig. 1C). Furthermore, we also failed to find sequence modifications at the predicted site in the *HBD* gene, despite close sequence similarity between *HBD* and *HBB* (Fig. 1D). These data suggest that the G1 gRNA to be a better candidate for further studies. Next, we synthesized a ssDNA oligo donor template that encoded 6 silent mutations and transfected this oligo alone or together with the G1 gRNA-Cas9 plasmid into 293T cells (Fig. 1E). We then extracted genomic DNA from the cells 48 h later for PCR amplification of the G1 target region. The PCR products were subsequently subcloned for sequencing. Compared to none from oligo-only control, analysis of 29 independent clones revealed 14 clones (48.3%) that perfectly matched the donor oligo template (Fig. 1E), indicating high efficiency of our approach and precise editing of the *HBB* locus in cells.

### CRISPR/Cas9-mediated editing of *HBB* gene in human tripronuclear zygotes

**Figure 2:**
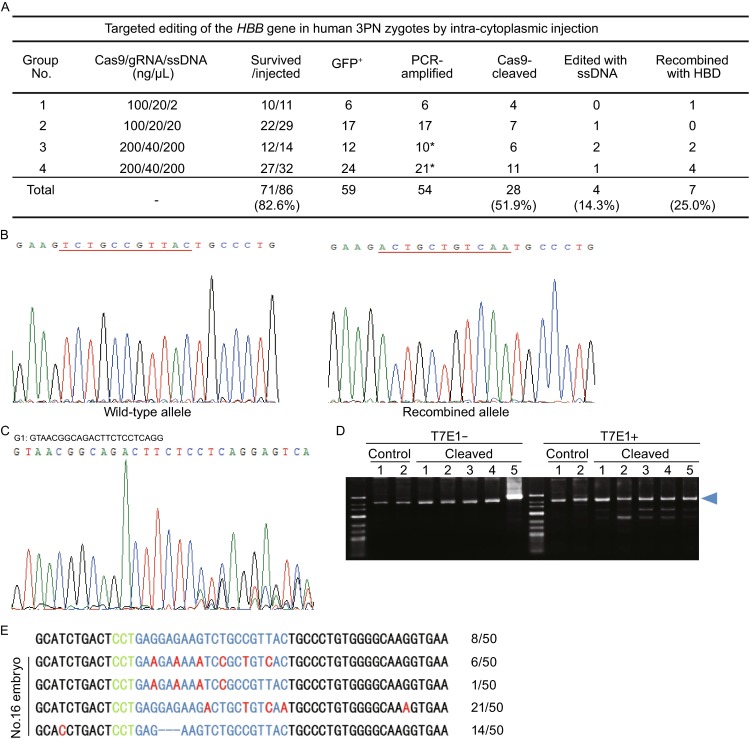
**Targeting of the** ***HBB*** **gene in human tripronuclear (3PN) zygotes using CRISPR/Cas9**. (A) Four groups of 3PN zygotes were injected intra-cytoplasmically with GFP mRNA (50 ng/μL) and Cas9/gRNA/ssDNA in different concentration combinations. The genomes of GFP^+^ embryos were first amplified by multiplex displacement amplification. The region spanning the target site was then PCR amplified, subcloned into TA vectors, and sequenced. * Indicates that target fragments in 5 GFP^+^ embryos failed to be PCR amplified. (B) Sequencing chromatographs of the wild-type allele and recombined allele generated by homologous recombination between *HBB* and *HBD* are shown here. The region with base substitution is underlined with red line. (C) A representative sequencing chromatogram of the region spanning the target site in Cas9-cleaved 3PN embryos. Double peaks near the PAM sequence (green) are indicated. (D) Five embryos with double peaks near the PAM sequence were randomly selected for the T7E1 assay. Blue arrowhead indicates the expected size for uncut PCR products. Control, amplified products from target regions with no double peaks near the PAM sequence. (E) Embryo No.16 from group 3 was used to PCR amplify sequences spanning the gRNA target regions of the *HBB* gene. The PCR products were then subcloned and sequenced. A total of 50 clones were examined, and the number of clones for each pattern indicated. PAM, green. G1 gRNA sequence, blue. Point mutations, red

To investigate the specificity and efficacy of gene targeting in human tripronuclear (3PN) zygotes, we co-injected G1 gRNA, Cas9 mRNA, GFP mRNA, and the ssDNA oligo into the cytoplasm of human 3PN zygotes in different concentration combinations (Fig. 2A). Based on morphology, ~80% of the embryos remained viable 48 h after injection (Fig. 2A), in agreement with low toxicity of Cas9 injection in mouse embryos (Wang et al., 2013; Yang et al., 2013). All GFP-positive embryos were then collected for whole-genome amplification by multiplex displacement amplification (Dean et al., 2002; Hosono et al., 2003), followed by PCR amplification of the G1 gRNA target region and sequencing. Of the 54 PCR-amplified embryos, 28 were cleaved by Cas9, indicating an efficiency of ~52% (Fig. 2A). Furthermore, 4 of the 28 Cas9-cleaved embryos (14.3%) were clearly edited using the ssDNA oligo as a repair template (Fig. 2A). Additionally, 7 embryos contained four identical point mutations in tandem, an clear indication of HDR using the *HBD* gene as a repair template (Fig. 2A and 2B). This finding suggests recombination of the *HBB* gene with *HBD* in 7 out of the 28 cleaved embryos (25%), even in the presence of co-injected exogenous ssDNA donor template (Fig. 2A and 2B). Similar observations have been found in mouse embryos, where endogenous homologous templates were found to compete with ssDNA oligos for HDR repair (Wu et al., 2013).

Because of the preference for the error-prone NHEJ pathway, the *HBB* sequences from Cas9-cleaved embryos showed double peaks near the PAM site on sequencing chromatographs (Fig. 2C). Analysis of 5 of these embryos using the T7E1 assay also confirmed successful cleavage by G1 gRNA and Cas9 (Fig. 2D). In addition, the gene-edited embryos were mosaic. For example, embryo No. 16 contained many different kinds of alleles (Fig. 2E).

### CRISPR/Cas9 has off-target effect in human tripronuclear embryos

**Figure 3:**
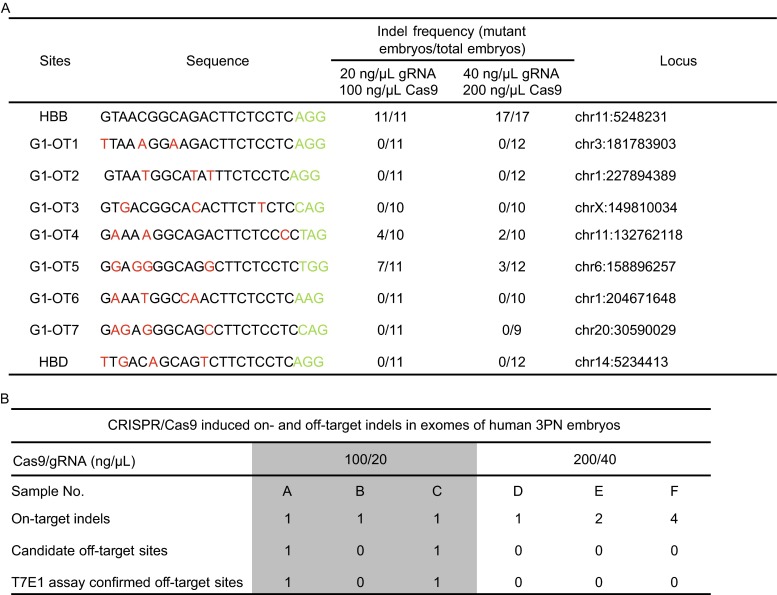
**Off-target cleavage of CRISPR/Cas9 in human 3PN embryos**. (A) Off-target cleavage in human embryos was summarized here. PAM sequence are labeled in green.* HBB*, on-target cleavage of the* HBB* locus. OT1–7, the top 7 predicted off-target sites. HBD, the predicted off-target site in the HBD locus. Mismatched nucleotides compared to the* HBB* locus are labeled in red. Some of the off-target sites failed to be amplified by PCR in this experiment. (B) Six Cas9-cleaved embryos were randomly selected (three each from groups 2 and 3) for whole-exome sequencing. Concentrations of the Cas9/gRNAs used for injections are indicated. Candidate off-target sites were also confirmed by T7E1 assay

To determine the off-target effects of CRISPR/Cas9 in these embryos, we again examined the top 7 potential off-target sites plus the site in the *HBD* gene. The T7E1 assay revealed off-target cleavage in the *OPCML* intron (G1-OT4) and the *TULP1* intron (G1-OT5) (Figs. 3A, S3 and S4), although none of these sites appeared to be cleaved in human 293T cells (Fig. 1C). We then randomly selected 6 *HBB*-cleaved embryos (three each from groups 2 and 3, Fig. 2A) for whole-exome sequencing. As shown in Fig. 3B, on-target indels were identified in all of the samples. Two candidate off-target sites within exons were found, where lower concentration of the Cas9 mRNA and gRNA had been used (sample A and C, Fig. 3B), and further confirmed through the T7E1 assay (Fig. S5). These two sites reside in the exons of the *C1QC* and Transthyretin (*TTR*) gene, both of which closely match the G1 gRNA sequence in the seed region (Fig. S6). These data demonstrate that CRISPR/Cas9 has notable off-target effects in human 3PN embryos.

### HDR of double strand breaks at the *HBB* gene occurs preferentially through the non-crossover pathway

**Figure 4:**
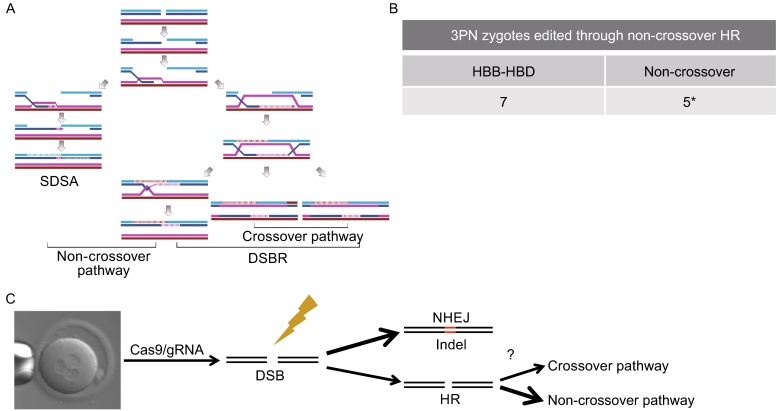
**Repair of double-strand breaks at the** ***HBB*** **gene in human early embryos occurs preferentially through the non-crossover pathway when HDR is utilized**. (A) In human cells, DSBs may be repaired through the double-strand break repair (DSBR) pathway or the non-crossover synthesis-dependent strand annealing (SDSA) pathway. Both crossover and non-crossover DSBR can occur. (B) The *HBD* locus from the 7 recombined 3PN embryos were similarly examined as above. * Indicates that the *HBD* locus failed to be amplified in two of the embryos. (C) In human embryos, repair of DSBs generated by CRISPR/Cas9 occurs mainly through NHEJ. If HDR is utilized, the non-crossover pathway is preferred

DSBs can be repaired through either error-prone NHEJ or high-fidelity HDR (Ciccia and Elledge, 2010; Moynahan and Jasin, 2010). There are three options for the HDR pathway, non-crossover synthesis-dependent strand annealing (SDSA), non-crossover double-strand break repair (DSBR), and crossover DSBR (Fig. 4A). Bi-directional sequence exchange between the recombined genes occurs with crossover, while uni-directional sequence exchange occurs in absence of crossover. Of the 3PN embryos examined thus far, 4 were repaired using the ssDNA oligo as template and 7 were recombined with the endogenous *HBD* gene (Fig. 2A). When the *HB*D locus from the 7 recombined 3PN embryos were amplified and examined, we found that the *HBD* locus in the 5 successfully-amplified embryos remained intact, containing no *HBB* sequences (Fig. 4B). This lack of bi-directional sequence exchange supports the notion that the *HBB* gene was repaired primarily through non-crossover HDR (San Filippo et al., 2008). It is possible that one of the alleles in 3PN embryo No.16 (group 3) (Fig. 2E), which only contained 4 of the 6 silent mutations from the ssDNA oligo, might have been generated by non-crossover pathway as well (Fig. 4A). Taken together, our results suggest that homologous recombination in human early embryos preferentially occur through the non-crossover HDR pathway (Fig. 4C), similar to what has been observed in human iPS cells (Byrne et al., 2014).

## DISCUSSION

In this study, we used 3PN zygotes to investigate the specificity and fidelity of the CRISPR/Cas9 system. Similar to cultured human cells, most of the DSBs generated by Cas9 in 3PN zygotes were also repaired through NHEJ (Fig. 2A). ssDNA-mediated editing occurred only in 4 embryos (14.3%), and the edited embryos were mosaic, similar to findings in other model systems (Shen et al., 2013; Yang et al., 2013; Yen et al., 2014). Endogenous homologous sequences were also used as HDR templates, with an estimated editing efficiency of 25% (Fig. 2A). This high rate of repair using endogenous sequences presents obvious obstacles to gene therapy strategies using CRISPR/Cas9, as pseudogenes and paralogs may effectively compete with exogenous templates (or endogenous wild-type sequences) during HDR, leading to unwanted mutations (Fig. 2B).

Our whole-exome sequencing result only covered a fraction of the genome and likely underestimated the off-target effects in human 3PN zygotes. In fact, we found that even with an 8 bp mismatch between the G1 gRNA and *C1QC* gene (Fig. S6), the CRISPR/Cas9 system was still able to target the *C1QC* locus in human 3PN embryos (Figs. 3B and S5). Such off-target activities are similar to what was observed in human cancer cells. Because the edited embryos are genetically mosaic, it would be impossible to predict gene editing outcomes through pre-implantation genetic diagnosis (PGD). Our study underscores the challenges facing clinical applications of CRISPR/Cas9.

Further investigation of the molecular mechanisms of CRISPR/Cas9-mediated gene editing in human model is sorely needed. In particular, off-target effect of CRISPR/Cas9 should be investigated thoroughly before any clinical application (Baltimore et al., 2015; Cyranoski, 2015; Lanphier et al., 2015).

## MATERIALS AND METHODS

### Construction and use of CRISPR plasmids

pX330 (Addgene, #42230) was used for transient transfection and pDR274 (Addgene, #42250) was used for *in vitro* transcription. We amplified the sequences encoding 3×Flag-tagged hCas9 from pX330 and cloned it into the *Not*I/*Age*I restriction sites of pDR274 to obtain pT7-3×Flag-hCas9. The pT7-3×Flag-hCas9 plasmid was linearized with PmeI and *in vitro* transcribed using the mMESSAGE mMACHINE T7 ULTRA kit (Life Technologies). The pDR274 vector encoding gRNA sequences was *in vitro* transcribed using the MEGAshortscript T7 kit (Life Technologies). The Cas9 mRNA and the gRNAs were subsequently purified with the MEGAclear kit (Life Technologies), resuspended in RNase-free water, and quantified using NanoDrop-1000.

Sequences for cloning the G1, G2, and G3 gRNAs into the pX330 vector are: pX330-G1-FP: CACCGTAACGGCAGACTTCTCCTC; pX330-G1-RP: AAACGAGGAGAAGTCTGCCGTTAC; pX330-G2-FP: CACCGTCTGCCGTTACTGCCCTGT; pX330-G2-RP: AAACACAGGGCAGTAACGGCAGAC; pX330-G3-FP: CACCGGCTGCTGGTGGTCTACCCT; pX330-G3-RP: AAACAGGGTAGACCACCAGCAGCC; Sequences for cloning the G1 gRNA into pDR274 are: pDR274-G1-FP: TAGGTAACGGCAGACTTCTCCTC; pDR274-G1-RP: AAACGAGGAGAAGTCTGCCGTTA.

The sequence for the ssDNA oligo used to repair* HBB* is: 5′-CAACCTGCCCAGGGCCTCACCACCAACTTCATCCACGTTCACCTTGCCCCACAGGGCAGTGACAGCGGATTTTTCTTCAGGAGTCAGATGCACCATGGTGTCTGTTTGAGGTTGCTAGTGAACAC-3′

### Identification and collection of human tripronuclear (3PN) embryos

Mature oocytes were inseminated in fertilization medium (Vitrolife, Sweden) 4 h after retrieval by conventional *in vitro* fertilization (IVF). Fertilization status was checked 16–19 h after insemination and normal fertilization was assessed by the presence of two clear pronuclei. Abnormal fertilized oocytes with three clear pronuclear were selected for cryopreservation.

### Embryo vitrification and recovery

Embryos were selected for cryopreservation using the CryoTop device as reported (Kuwayama et al., 2005). Briefly, embryos were incubated in Vitrification Solution 1 (7.5% (*v*/*v*) DMSO (*v*/*v*) and 7.5% (*v*/*v*) ethylene glycol) for 5–6 min, and then moved to Vitrification Solution 2 (15% (*v*/*v*) DMSO, 15% (*v*/*v*) ethylene glycol, and 0.65 mol/L sucrose) for 30 s. The embryos were then quickly placed onto a Cryotop (Kitazato Supply Co., Fujinomiya, Japan), followed by aspiration of excess medium with a fine pipette and quick immersion in liquid nitrogen. The embryos were then stored in liquid nitrogen. For recovery, the embryos were warmed with the polypropylene strip of the Cryotop immersed directly into 3 mL of 1.0 mol/L sucrose at 37°C for 1 min, retrieved and held for 3 min in 1 mL of a dilution solution (0.5 mol/L sucrose in TCM199 medium with 20% serum substitute supplement), and then washed at room temperature before being cultured for subsequent analysis.

### Analysis of CRISPR/Cas9 induced cleavages

The T7 endonuclease 1 (T7E1) cleavage assay was performed as described by Shen et al. (Shen et al., 2014). For verification of indels and mutations, genomic DNA was used for PCR amplification of target sites with primers listed in Supplementary information, Table S1. PCR products were sequenced directly using primers from Supplementary information, Table S1 to confirm the presence of double peaks, and those with double peaks were then TA cloned into the pGEM-T vector (Promega) for sequencing. In general, a total of 45–50 clones were sequenced for each embryo.

To identify potential off-target sites, we used the online tool (http://crispr.mit.edu/). Sequences surrounding these genomic sites were PCR amplified for the T7E1 assay with primers listed in Table S1.

### Whole genome amplification using embryos

Whole genome amplification of the embryos was performed using the PEPLI-g Midi Kit (Qiagen). Briefly, embryos were transferred into PCR tubes containing reconstituted buffer D2 (7 μL), and then incubated at 65°C for 10 min, before the addition of Stop solution (3.5 μL) and MDA master mix (40 μL) and incubation at 30°C for 8 h. The DNA preparation was diluted with ddH_2_O (3:100), and 1 μL of the diluted DNA was used for PCR analysis.

### Whole-exome sequencing, data processing, and off-target analysis

The exome was captured using the 50 Mb SureSelectXT Human All Exon V5 kit (Agilent). The enriched exome was sequenced on Illumina HiSeq 2000 PE100 as paired-end 100 bp reads, which were aligned to the human reference genome (UCSC, hg19) by means of BWA with default parameters (v0.7.5a) (Li and Durbin, 2010). Samtools (v0.1.19, http://samtools.sourceforge.net) and Picard tools (version 1.102, http://picard.sourceforge.net) were used to build indices and remove duplicates. Local realignment around indels (RealignerTargetCreator, IndelRealigner) and base score recalibration (BaseRecalibrator) were applied by GATK (The Genome Analysis ToolKit, version 3.1-1) (McKenna et al., 2010) to ensure accuracy in identifying indels and single nucleotide variants (SNVs). GATK HaplotypeCaller and Samtools were used to call variants for six samples and the union variants of both obtained by CombineVariants were then divided into indels and SNVs by SelectVariants.

We first excluded indels and SNVs located outside of exon regions following annotation by ANNVAR based on RefSeq gene models (hg19) (Wang et al., 2010). A total of 7463 indels and 188,078 SNVs passed this filter. Next, indels and SNVs with more than two reads were retained by VariantFiltration and Python, discarding low-quality and unlikely indels (8.99%) and SNVs (5.91%).

To avoid false positive calls that overlap with repeat sequences and/or include homopolymers (Bansal and Libiger, 2011), we removed indels and SNVs that overlapped with low-complexity regions as defined by RepeatMasker (UCSC Genome Browser) and filtered out indels and SNVs containing homopolymers (>7 bp) in the low-complexity flanking region (±100 bp), removing 55.58% of potential indels and 17.01% of potential SNVs. To more definitively assign indels, we searched the ±100 bp regions flanking the potential indel sites for potential off-target sites. Bowtie1 (version 0.12.8, http://bowtie-bio.sourceforge.net) was used to align gRNA sequences (20 bp) to the ±100 bp sequences, allowing for ≤6 mismatches or perfect match of the last 10 nt 3′ of the gRNA. Successfully aligned sites with an NRG PAM were deemed on/off-target sites. Of the 12 candidate indels identified by this analysis, there were ten on-target indels in all samples and two off-target indels in samples A and C. Candidate off-target sites were further confirmed by PCR and sequencing. The results are summarized in Table S2.

## Electronic supplementary material

Supplementary material 1 (PDF 617 kb)
