# Supplementary material for: CRISPR/Cas9-mediated gene editing in human tripronuclear zygotes
Source: Protein Cell. 2015 Apr 18;6(5):363–72. doi: 10.1007/s13238-015-0153-5 (PMC4417674; doi:10.1007/s13238-015-0153-5)
Supplement: Supplementary file 1 — Supplementary material 1 (PDF 617 kb) [file 13238_2015_153_MOESM1_ESM.pdf]

Supplementary information for:

CRISPR/Cas9-mediated gene editing in human tripronuclear zygotes

Puping Liang*, Yanwen Xu*, Xiya Zhang*, Chenhui Ding*, Rui Huang, Zhen Zhang, Jie Lv, Xiaowei Xie, Yuxi Chen, Yujing Li, Ying Sun, Yaofu Bai, Zhou Songyang, Wenbin Ma, Canquan Zhou✉, Junjiu Huang✉

Guangdong Province Key Laboratory of Reproductive Medicine, the First Affiliated Hospital, and Key Laboratory of Gene Engineering of the Ministry of Education, School of Life Sciences, Sun Yat-sen University, Guangzhou 510275, China

*Puping Liang, Yanwen Xu, Xiya Zhang and Chenhui Ding have contributed equally to this work.

✉Correspondence: hjunjiu@mail.sysu.edu.cn (J. Huang), zhoucanquan@hotmail.com (C. Zhou)

Supplementary figures

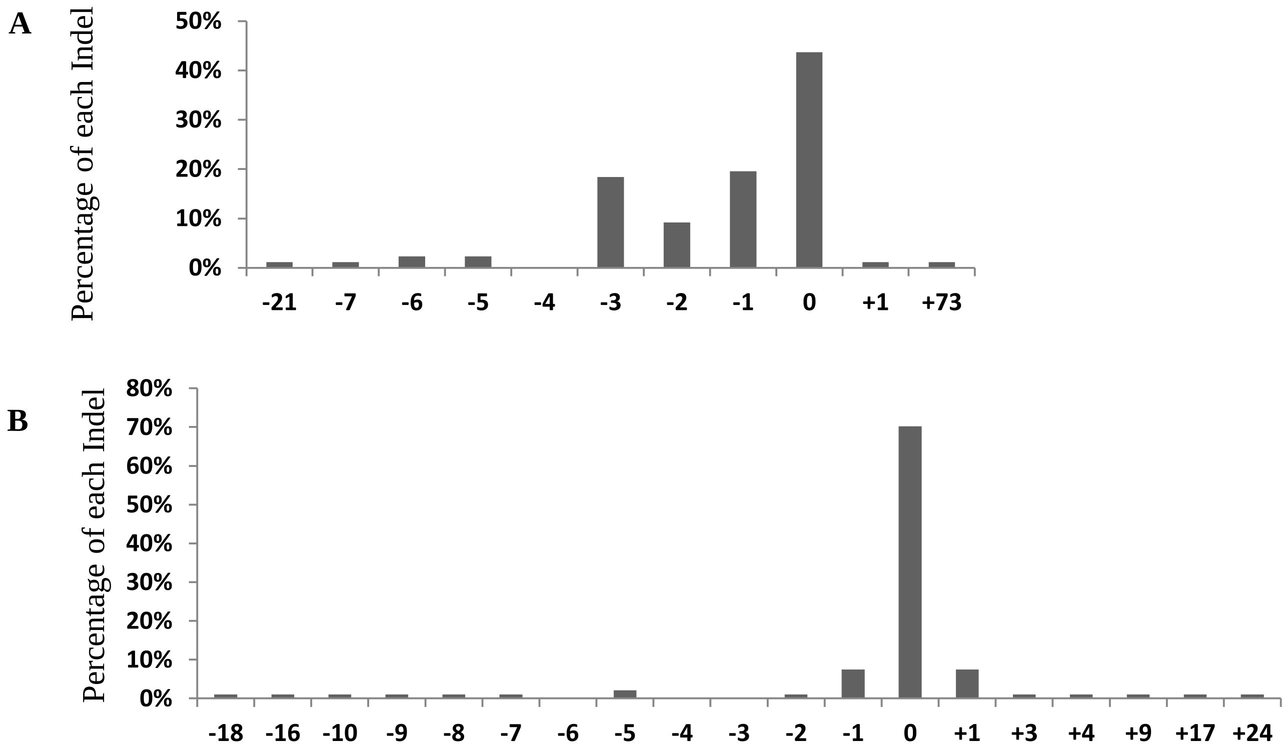

Supplementary Fig 1. Indel spectra of G1 and G2 gRNAs. 293T cells were transfected with 2µg of pX330-G1 **(A)** or pX330-G2 (**B**). The region spanning each target site was PCR amplified. And the PCR products were subcloned into TA vectors and sequenced. About 50 clones were sequenced, and the results are summarized here. The size of each indel was calculated. The y-axis shows the percentage of each indel.

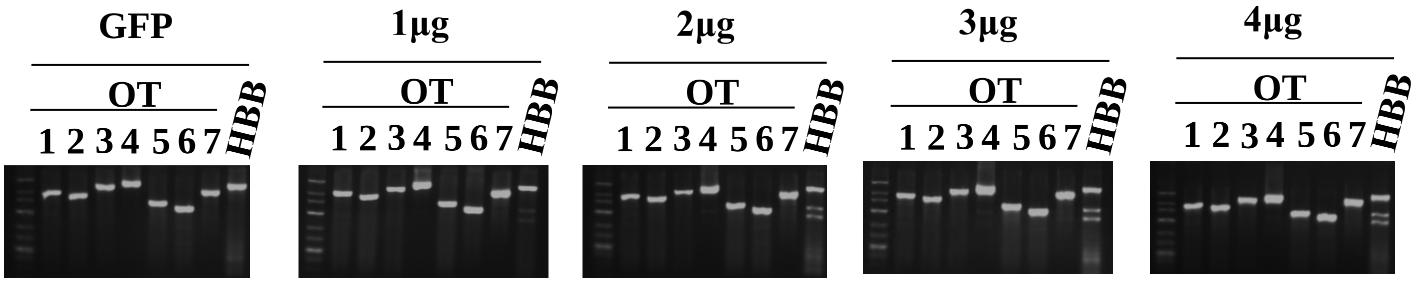

Supplementary Fig 2. G2 gRNA showed detectable off-target cleavage at OT-4 site. 293T cells were transfected with increasing concentrations (1μg, 2μg, 3μg, 4μg) of the G2 gRNA-Cas9 vector. A GFP expression vector was used as transfection control. Regions spanning the top 7 predicted off-target sites for each gRNA were PCR amplified for the T7E1 assay. OT, off-target. HBB, on-target editing in the HBB gene locus.

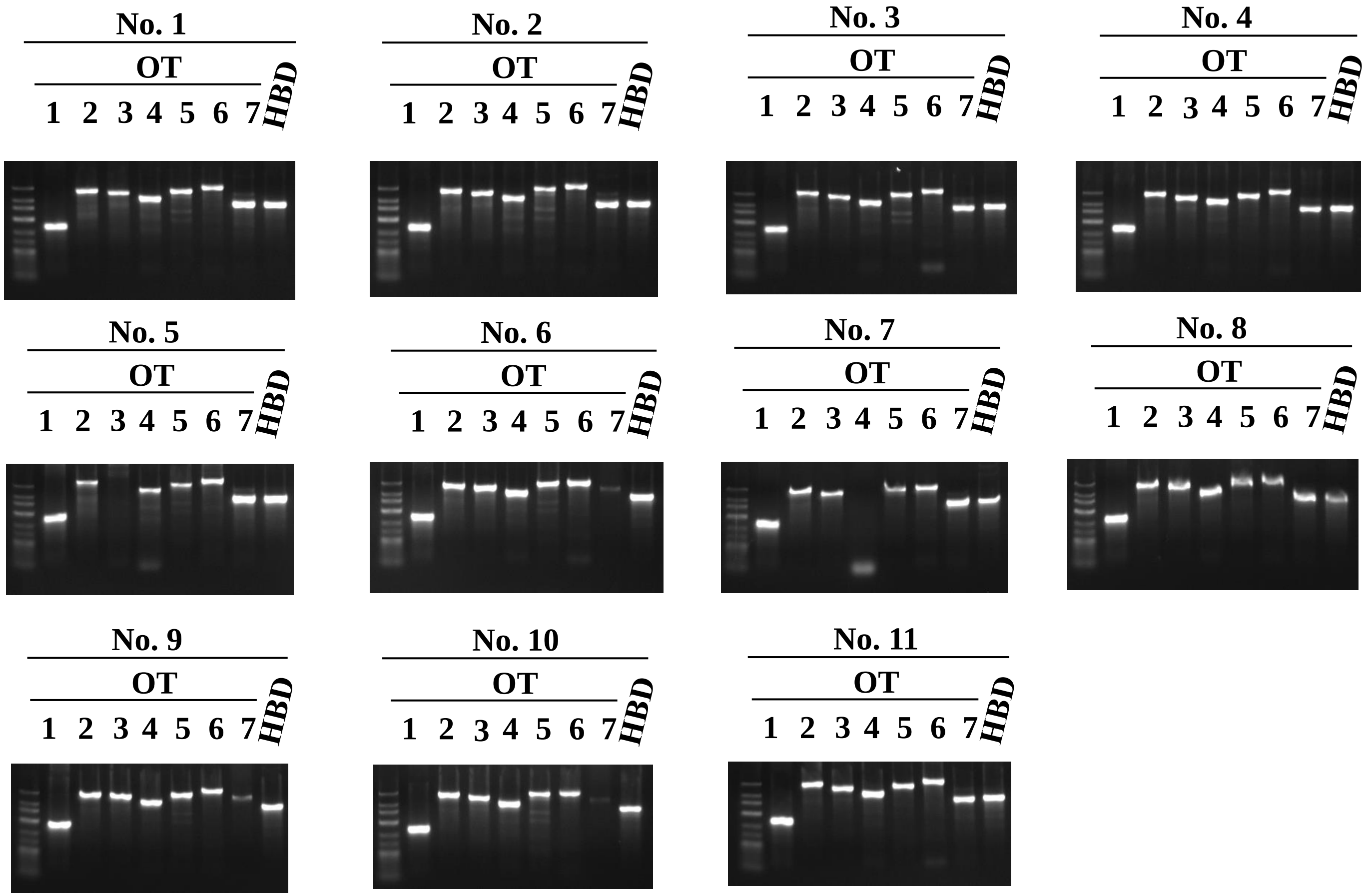

Supplementary Fig 3. Assessment of off-target cleavage in human 3PN embryos in the low concentration (100/20) group by the T7E1 assay. Seven predicted potential off-target (OT) sites and the site in *HBD* locus were amplified by PCR for the T7E1 assay to detect CRISPR/Cas9–mediated cleavage. The smaller bands in each lane indicate off-target cleavage.

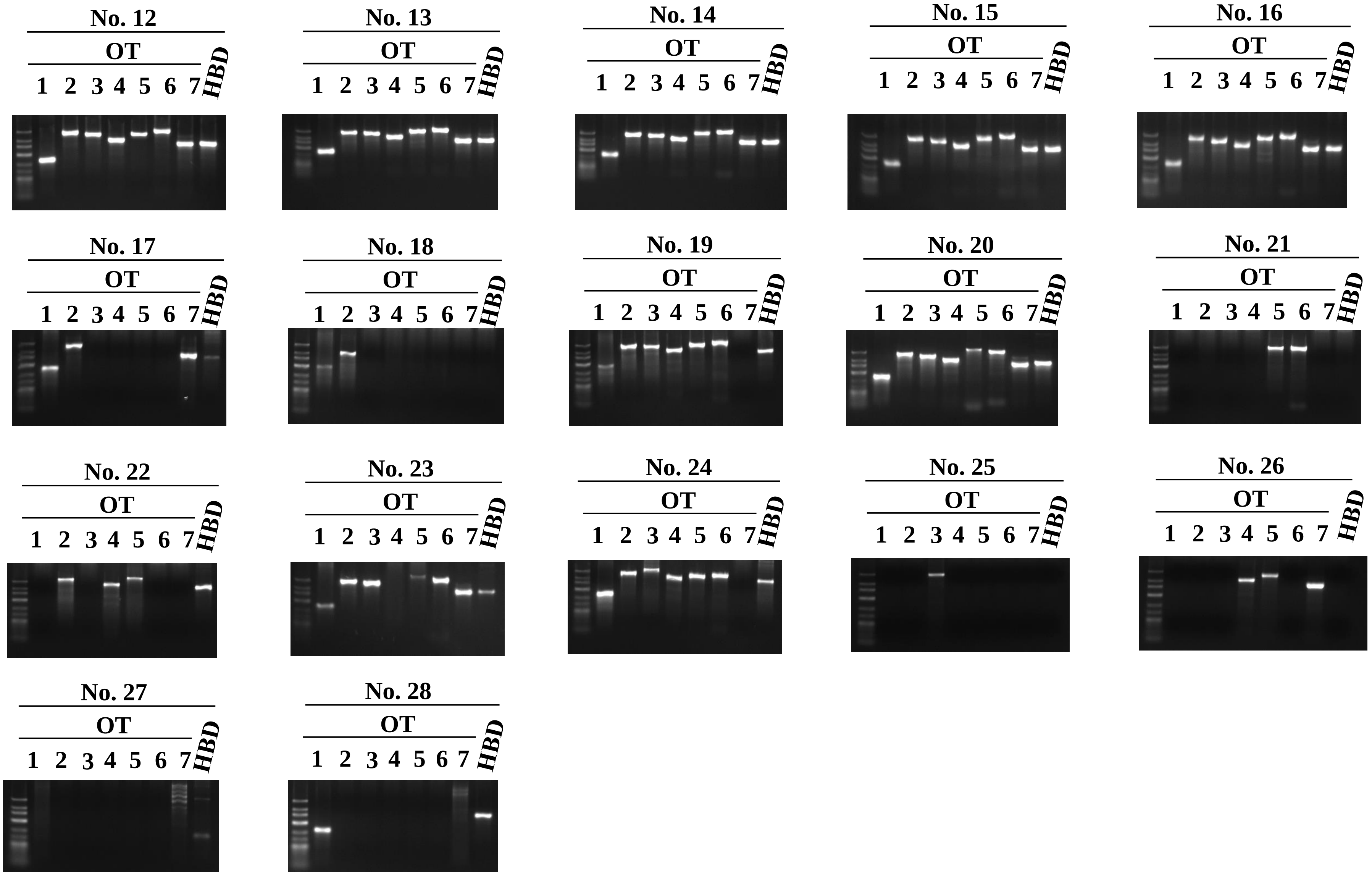

Supplementary Fig 4. Assessment of off-target cleavage in human 3PN embryos in the high concentration (200/40) group by the T7E1 assay. Eight potential off-target sites were amplified by PCR for the T7E1 assay. The smaller bands in each lane indicate off-target cleavage. Some of the target sites failed to be amplified in some samples.

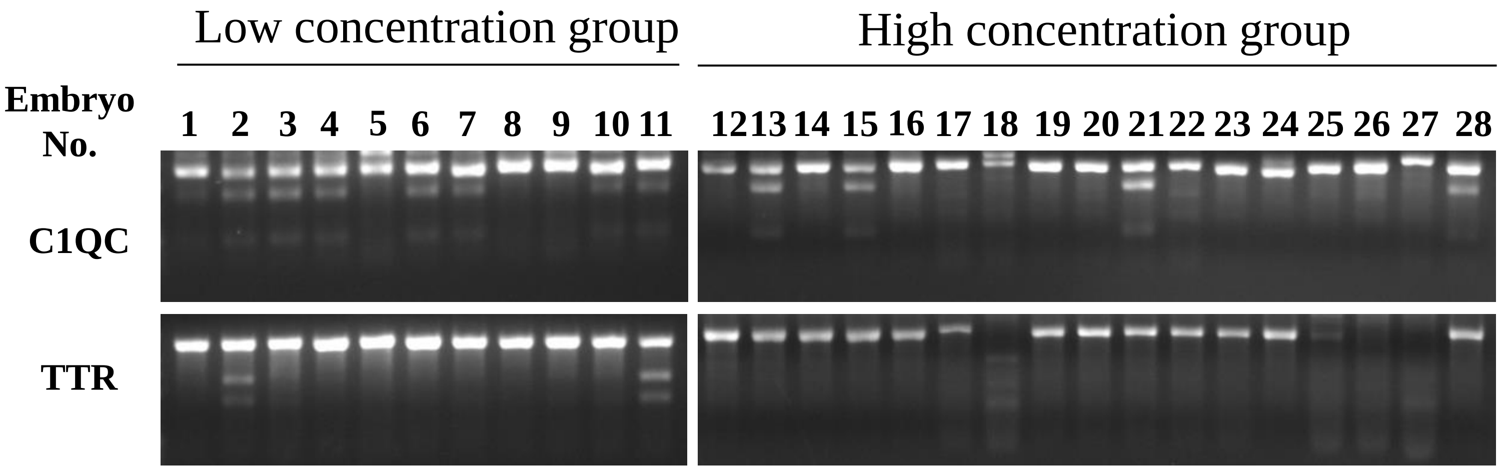

Supplementary Fig 5. **Confirmation of off-target cleavage in the C1QC and TTR loci in human 3PN embryos by the T7E1 assay**. Two candidate off-target sites, detected by exome sequencing, were amplified by PCR for the T7E1 assay to detect CRISPR/Cas9–mediated cleavage. The smaller bands in each lane indicate off-target cleavage. Some of the target sites failed to be amplified in some samples.

GTAACGGCAGACTTCTCCTCagg	gRNA-G1 target sequence
GGAATGAGGGACTTCTCCTCcag	Off-target site on TTR
CACCAGATGGACTTCTCCTCcag	Off-target site on C1QC

Supplementary Fig 6. The off-target sites in the *TTR* gene and *C1QC* gene.
PAM, green. Mismatched nucleotides, red.

Supplementary Table 1. List of PCR primers to amplify genomic regions, assess editing status, and examine off-target sites for G1 and G2 gRNAs.

Primers	Locus	Direction	Sequence (5' to 3')
HBB-T7E1	chr11:5248231	F	AGTCCAACTCCTAAGCCAGTG
		R	GAGGTTGTCCAGGTGAGCC
G1-OT1	chr3:181783903	F	TGTCAAGGTTTATGAGAGGTCTG
		R	CATGTGTTCTGTGTATGTGTGTGTA
G1-OT2	chr1:227894389	F	AGAGGGGGCTGACACGTTA
		R	TTTGTGTTCTCATGATGCAGCG
G1-OT3	chrX:149810034	F	GGTTCCGTATCGTGCCTCA
		R	GAACTAGGTGCAGTGATACCGT
G1-OT4	chr11:132762118	F	CCCTATACCTGGGCTCCGTT
		R	GAAAGGGCCTCTCTCTTTGTAATG
G1-OT5	chr6:158896257	F	AAGCTCTACAAGGGCAGAGAATG
		R	TCAAAGCTCCCAGATTCACGTT
G1-OT6	chr1:204671648	F	GGCTCTAGGTGAGCTTGTGG
		R	CCCACCACACTGTCAGTACC
G1-OT7	chr20:30590029	F	CTGAGACCTGGGGCTGGG
		R	TGGGGGGATTTGGGTGAG
HBD- T7E1	chr11: 5234396	F	AGAACAGCCAATCTCAGGG
		R	CCAAGGGTAGACCACCAGTA
G2-OT1	chr9:104595866	F	CGAAATGATTGGAACCATGGGA
		R	CCTCCAGTTTCTAAGAGCGGTG
G2-OT2	chr6:157157457	F	AGGTAACAGTCGACGTCAGTA
		R	CTTTAACAGGCAAGGACTCAACC
G2-OT3	chr2:179603682	F	ACCATGCTGAATGGGAACACT
		R	TGAGGCCTTGAATGACAGCG
G2-OT4	chr8:568439	F	TGCCCTATGCGTGCTCACT
		R	GTAATGTTGCCCAAGGTCTCTG
G2-OT5	chr4:18681607	F	CAGGAGCTTCCCTTCACAGA
		R	TGGGCAGCAGGAATGAATGA
G2-OT6	chr4:88057690	F	ATTGCCTAGAGCGCTGCAC
		R	TGGCTGGACAACATGAGTTACC
G2-OT7	chr19:1379107	F	CTGGGGAGGCTTAGATGGGA
		R	AAAGCGTGCAGGCTTCTGAG

Supplementary Table 2. Summary of Indels and Single Nucleotide Variants (SNVs) Detected by Exome Sequencing

	A	B	C	D	E	F
Raw indel	32,546	39,506	37,707	40,919	36,355	41279
Indel in exon	1,182	1,267	1,180	1,327	1,232	1275
Reads number >2 indel	1,091	1,131	1,085	1,199	1,133	1153
Post Low-complexity filter	698	767	712	795	739	740
Post Homopolymeric filter	487	521	489	517	487	516
F1anking region with potential off-target sites indel	2	1	2	1	2	4
On-target indels	1	1	1	1	2	4
Candidate off-target indels	1	-	1	-	-	0
sample-specific indels	1	-	1	-	-	0
Raw SNV	319,448	379,535	353,982	389,704	356,904	395349
SNV in exon	30,987	30,444	30,168	32,701	32,126	31652
Reads number >2 SNV	29,178	28,531	28,512	30,838	30,202	29699
Post Low-complexity filter	25,979	25,146	25,291	27,373	26,854	26256
Post Homopolymeric filter	24,319	23,513	23,668	25,628	25,158	24572
F1anking region with potential off-target sites SNV	19	15	24	19	25	23
sample-specific SNVs	3	1	9	2	9	7
